# Hot-water immersion increases popliteal artery shear stress in Peripheral Arterial Disease

**DOI:** 10.1186/2046-7648-4-S1-A111

**Published:** 2015-09-14

**Authors:** Kate N Thomas, Andre M Van Rij, Samuel JE Lucas, James D Cotter

**Affiliations:** 1 Department of Surgical Sciences, University of Otago, Dunedin, New Zealand; 2 Department of Physiology, University of Otago, Dunedin, New Zealand; 3 School of Physical Education, Sport and Exercise Sciences, University of Otago, Dunedin, New Zealand; 4 School of Sport, Exercise and Rehabilitation Sciences, University of Birmingham, Birmingham, UK

## Introduction

Exercise improves peripheral vascular function in healthy and diseased populations; this is partly attributed to the exposure of the endothelium to transient, repetitive increases in blood flow and antegrade shear stress [1]. Traditional exercise poses significant barriers for patients with Peripheral Arterial Disease (PAD), as the condition manifests as debilitating walking-induced muscle pain (claudication) caused by obstruction to blood flow. PAD patients represent a large cohort who might therefore benefit from an alternative approach to exercise. Local limb heating in young, healthy adults has been shown to induce blood flow patterns indicative of promoting beneficial adaptations in peripheral arteries [2-4]. Additionally, preliminary evidence exists demonstrating sauna therapy improves symptoms and clinical indices in PAD [5]. The aim of this study was to investigate the acute vascular and cardiovascular responses to heat by lower limb hot-water immersion in patients with PAD and in healthy, elderly controls.

## Methods

Eight patients with PAD (6 male, age 69 ± 5 y) and nine controls free from PAD (8 male, age 71 ± 6 y) underwent hot-water immersion (30 min immersed to the waist in water at 42-43 °C). Using high-resolution ultrasound, the popliteal artery diameter and blood flow was assessed before, during the last 3 min and 30 min after immersion in order to calculate the shear stress stimulus on the endothelium. Blood pressure and heart rate were continuously assessed using finger photoplethysmography.

## Results

Antegrade popliteal shear rate increased more than three-fold during water immersion in both PAD (mean ± SD: +112 ± 79 s^-1^, *p *< 0.01) and controls (+74 ± 51 s^-1^, *p *< 0.01). At 30 min after immersion, shear rate remained elevated above baseline levels, although not significantly so for PAD (PAD: +25 ± 33 s^-1^, *p *= 0.09 vs. baseline, controls: +15 ± 14 s^-1^, *p *= 0.01). Retrograde shear was absent in PAD throughout but significantly decreased during immersion in controls (-8 ± 5 s^-1^, *p *< 0.01). Systolic (SBP) and diastolic blood pressure (DBP) were reduced (*p *< 0.001) during water immersion in PAD and controls to a similar extent: SBP in PAD, -36 ± 21 mm Hg, controls, -32 ± 13 mm Hg; DBP, -14 ± 7 and -12 ± 8 mm Hg, respectively (*p *> 0.05 for interaction effects). SBP remained lower than baseline in both groups 30 min after immersion (*p *< 0.01). Heart rate increased similarly between groups, by 19 ± 11 beats.min^-1 ^in PAD and 27 ± 14 beats.min^-1 ^in controls (time: *p *< 0.001; interaction: *p *= 0.16).

## Discussion

A single bout of hot-water immersion induced favourable shear stress patterns in the popliteal artery of PAD patients and healthy, elderly controls. This heat stress also induced a marked blood pressure-lowering effect, valuable particularly in a PAD population, who are commonly hypertensive yet unable to exploit the post-exercise hypotensive effect.

## Conclusion

Considering the shear stress and blood pressure effects demonstrated in this study, heat stress, if repeated, has potential to result in beneficial vascular adaptations for a group with limited access to exercise; although this remains to be confirmed.
